# Efferocytosis failure in chronic rhinosinusitis with nasal polyps: receptor-level mechanisms of unresolved type 2 inflammation

**DOI:** 10.3389/fimmu.2026.1938524

**Published:** 2026-08-13

**Authors:** Sihai Tang, Hongbing Liu

**Affiliations:** Department of Otolaryngology – Head and Neck Surgery, The Second Affiliated Hospital, Jiangxi Medical College, Nanchang University, Nanchang, Jiangxi, China

**Keywords:** apoptotic cell clearance, Axl, chronic rhinosinusitis with nasal polyps (CRSwNP), efferocytosis, eosinophilic inflammation, macrophage polarization, MERTK, resolution of inflammation

## Abstract

Chronic rhinosinusitis with nasal polyps (CRSwNP) is a prototypical type 2 airway disease in which tissue eosinophilia persists. That persistence is conventionally attributed to excess recruitment and to slowed apoptosis. Both explanations concern arrival and survival, and neither specifies what becomes of an eosinophil once it has died. That step is efferocytosis, the receptor-guided ingestion of apoptotic cells by macrophages, which differs from microbial phagocytosis in its ligands, its signaling output and its metabolic consequences. We establish this distinction first, because it decides which published datasets qualify as evidence and which do not. We then grade the surviving evidence and propose a cascade-anchored model in which failure is localized to three candidate lesions in the polyp macrophage: loss of an interleukin-10-dependent, MerTK-competent subset; a receptor repertoire skewed toward AXL and TIM-4 in which expression is uncoupled from productive ingestion; and an execution block at the phosphoinositide and Rac1 module. This model assigns the macrophage subsets reported in CRSwNP, including IL-10-deficient, ALOX15-positive, TIM-4-positive, TIPE2-positive, HMOX1-high and INPP4A-deficient populations, to defined steps rather than to a *polarization* label. We restate the shedding hypothesis as reduced surface receptor availability and reconcile a discordant tissue sheddase dataset. We explain why locally raised pro-resolving mediators cannot compensate when the receptor executing their action is absent, filter candidate therapies through the constraints of a colonized mucosal barrier, and note that the same receptors are targeted for inhibition in oncology. Finally, we specify the measurements that would refute the model.

## 1 Introduction

CRSwNP is best understood as a clinical phenotype layered onto several inflammatory endotypes, of which a type 2 endotype predominates in most European and North American cohorts and in a substantial proportion of Asian ones (1–3). Tissue eosinophilia carries prognostic weight for severity and for recurrence after surgery (4). Two mechanisms are conventionally invoked to explain why that eosinophilia persists: eosinophils are recruited in excess, and once recruited they die more slowly (5). Both statements describe arrival and survival. Neither specifies what becomes of an eosinophil once it has died.

That question is answered by efferocytosis, the receptor-guided ingestion of apoptotic cells by macrophages, which removes dying cells while their membranes remain intact and simultaneously instructs the phagocyte to adopt a resolving program (6, 7). When ingestion is too slow, apoptotic cells lose integrity and discharge contents that behave as danger signals, so the clearance step is what converts a self-limiting process into a self-sustaining one (8).

**Figure 1:**
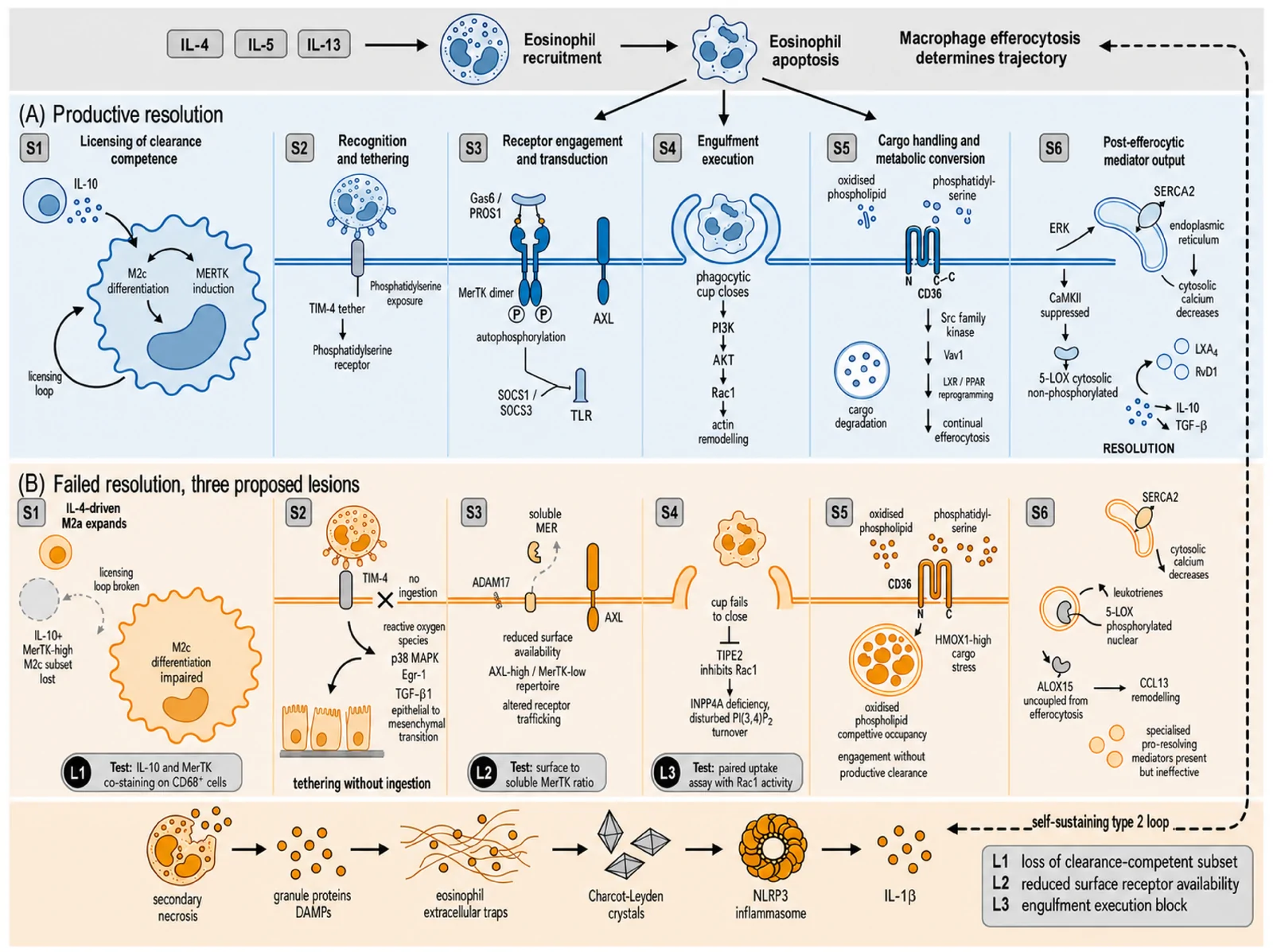
Efferocytic failure as a discrete mechanistic node between eosinophil apoptosis and persistent type 2 inflammation in chronic rhinosinusitis with nasal polyps. Both panels share an identical six-stage layout, S1 to S6, so that differences can be read by position. Type 2 cytokines recruit eosinophils, recruited eosinophils undergo apoptosis, and macrophage efferocytosis then determines the trajectory. **(A)** Productive resolution. Interleukin-10 licenses M2c differentiation and MERTK induction through a self-reinforcing loop (S1); externalised phosphatidylserine on the dying eosinophil is tethered by TIM-4 (S2) and engaged by a MerTK dimer through the bridging molecules Gas6 and PROS1, with autophosphorylation and SOCS1- and SOCS3-dependent restraint of Toll-like receptor *signaling* (S3); phosphoinositide 3-kinase, protein kinase B and Rac1 drive actin remodeling so that the phagocytic cup closes (S4); internalised cargo is degraded and CD36, drawn as a two-pass hairpin receptor with both termini intracellular, contributes to Src family kinase and Vav1 *signaling* and to liver X receptor and peroxisome proliferator-activated receptor reprogramming that permits continual efferocytosis (S5); and an extracellular signal-regulated kinase arm induces SERCA2 on the endoplasmic reticulum, lowering cytosolic calcium and suppressing calcium/calmodulin-dependent protein kinase II, thereby preserving the cytosolic non-phosphorylated pool of 5-lipoxygenase from which lipoxin A4 and resolvin D1 are generated alongside interleukin-10 and transforming growth factor β (S6). **(B)** Failed resolution, three proposed lesions. L1, loss of the interleukin-10-positive, MerTK-high M2c subset, shown as a faded dashed circle, with a broken licensing loop, impaired M2c differentiation and expansion of interleukin-4-driven M2a cells. L2, reduced surface receptor availability, comprising ADAM17-dependent release of soluble MER, an AXL-high and MerTK-low repertoire and altered receptor trafficking, together with TIM-4 tethering that proceeds not to ingestion but to reactive oxygen species, p38 mitogen-activated protein kinase, early growth response protein 1, transforming growth factor β1 and epithelial to mesenchymal transition. L3, an execution block at the phosphoinositide and Rac1 module, in which TIPE2 inhibits Rac1 and INPP4A deficiency disturbs phosphatidylinositol 3,4-bisphosphate turnover, so that the cup fails to close and the apoptotic eosinophil remains extracellular; downstream, oxidised phospholipid competitively occupies CD36 giving engagement without productive clearance, HMOX1-high cargo stress accumulates, 5-lipoxygenase becomes phosphorylated and nuclear with a shift toward leukotrienes, ALOX15 is uncoupled from efferocytosis and drives CCL13-dependent remodeling, and specialized pro-resolving mediators are present but ineffective. Badges mark the principal site of each lesion, while downstream consequences appear in adjacent stages, so that L2 encompasses the tethering defect at S2 as well as the receptor changes at S3, and L3 encompasses the cargo and mediator abnormalities at S5 and S6. Grey boxes beside each badge state the measurement that would test that lesion. In the lower band, uncleared eosinophils undergo secondary necrosis and release granule proteins, damage-associated molecular patterns, eosinophil extracellular traps and Charcot-Leyden crystals, which activate the NLRP3 inflammasome and interleukin-1β and re-amplify the type 2 circuit through the dashed feedback arrow. Pathways are distinguished by color, by line style and by label, so that the figure remains legible in grayscale. Lesion abbreviations are defined in the key at lower right.

We argue that fate control after eosinophil death, rather than eosinophil death itself, is rate limiting for resolution in eosinophilic CRSwNP, and that it fails at the level of macrophage receptors. The argument is built in a fixed sequence. Section 2 explains why efferocytic competence cannot be read off general phagocytic capacity, which determines which published datasets qualify as evidence. Section 3 grades the evidence that survives that filter. Section 4 proposes a cascade-anchored model with three candidate lesions and assigns the macrophage subsets reported in CRSwNP to defined steps (**Figure 1**; **Table 1**). Section 5 reconciles a discordant tissue dataset. Section 6 explains why raised pro-resolving mediators fail to compensate. Section 7 filters therapeutic proposals through sinonasal constraints, and Section 8 states what would refute the model. Literature was identified by searching PubMed and Web of Science through July 2026, combining terms for efferocytosis and phosphatidylserine receptors with terms for chronic rhinosinusitis, asthma and resolution of inflammation, supplemented by reference list screening; searches deliberately included terms for receptor inhibition in oncology, so that evidence pointing in the opposite therapeutic direction was represented rather than omitted.

**Table 1:** A cascade-anchored map of proposed efferocytic failure in chronic rhinosinusitis with nasal polyps: reported molecules, evidence tier, proposed point of failure, testable readout and therapeutic entry point.

Step	Molecules and signalling	Reported finding (setting; ref)	Tier	Proposed point of failure and consequence	Testable readout and therapeutic entry
S1 Licensing of clearance competence	IL-10 → M2c differentiation → MERTK induction	IL-10-positive M2 reduced in eosinophilic polyps, inversely related to symptom score and polyp size (CRSwNP; 27, 28); IL-10 required for MERTK induction (human MΦ; 26)	iii, ii	Selective loss of the MerTK-competent M2c-like subset while IL-4-driven M2a expands, so M2 marker positivity dissociates from clearance competence	IL-10 and MerTK co-staining on CD68-positive cells; IL-10-directed metabolic reprogramming (56) **[D]**
S2 Recognition and tethering	TIM-4, a non-catalytic PS tether; reactive oxygen species, p38, Egr-1 → TGF-β1	TIM-4 increased on sinonasal MΦ; neutralisation reduces polypoid lesions (CRSwNP and murine; 32)	iii	Tethering without ingestion where a partner kinase is limiting, producing profibrotic PS sensing instead of clearance	Binding versus uptake assay; TIM-4 neutralising antibody **[T]**
S3 Receptor engagement and transduction	MerTK via Gas6 or PROS1 → PI3K, AKT, Rac1; AXL under inflammatory or IL-4-conditioned states; TAM → SOCS1 and SOCS3	Mer deficiency delays resolution with uncleared eosinophils (murine; 14); airway MΦ AXL reduced (asthma; 16); AXL overexpression and aberrant TAM expression track severity and recurrence (CRSwNP; 19, 20)	i, ii, iii	Reduced surface availability through cleavage (40), altered trafficking or transcription, together with an AXL-high, MerTK-low repertoire	Surface to soluble MerTK ratio; soluble MER and AXL in lavage (42); sheddase inhibition or cleavage-resistant receptor **[R]**, given that TAM signalling restrains innate defence (9) and that the opposite direction is pursued in oncology (59, 60)
S4 Engulfment execution	Phosphoinositide turnover and Rac1 cycling; TIPE2, a phosphoinositide transfer protein inhibiting Rac; INPP4A hydrolysing PI(3,4)P_2_	TIPE2 raised in proportion to eosinophilia and severity (CRSwNP; 35, 36); INPP4A deficiency favours pathological M2 (CRSwNP; 37)	iii	Execution block downstream of intact binding, so receptor staining is preserved while uptake fails	Paired uptake assay with Rac1 activity; no agent available, target discovery required
S5 Cargo handling and metabolic conversion	CD36 reading PS and oxPL, with Src family kinases and Vav1; LXR and PPAR programmes; HMOX1	CD36 loss delays alveolar apoptotic cell clearance and lowers IL-4, IL-9, IL-13 and arginase 1 (murine; 39); HMOX1-high M2 track eosinophil chemokines (CRSwNP; 38)	i, iii	Competitive occupancy of CD36 by oxPL in an oxidant-rich polyp, giving engagement without productive clearance, plus cargo overload	oxPL competition assay in polyp MΦ; CD36 restoration **[D]**
S6 Post-efferocytic mediator output	MerTK, ERK, SERCA2, CaMKII → cytosolic 5-lipoxygenase → lipoxin A4 and resolvin D1; ALOX15 induced by efferocytosis via LXR	SPMs raised yet inflammation persists (CRSwNP; 43, 44); MerTK cleavage limits SPM biosynthesis (murine; 46); resolvin D1 acts by preventing MerTK loss (murine; 45); ALOX15-positive M2 drive CCL13 remodelling (CRSwNP; 33); LXR-dependent induction (human MΦ; 11, 34)	ii, i, iii	An intact mediator signal arriving at an absent receptor, with enzyme and product uncoupled so that flux favours remodelling over lipoxins	Ratio of pro-resolving to pro-inflammatory mediators; explant SPM response stratified by baseline surface MerTK (47) **[T]**

MΦ, macrophage; oxPL, oxidized phospholipid; PS, phosphatidylserine; SPM, specialized pro-resolving mediator.

Stages S1 to S6 correspond to those in **Figure 1**. Evidence tiers: i, causal demonstration *in vivo*; ii, functional measurement in human cells or tissue; iii, association in human CRSwNP tissue, with expression readouts only; iv, circumstantial or inferred. Feasibility at the sinonasal barrier: T, topically deliverable; D, delivery limited by mucus, biofilm or oedema; R, carrying antimicrobial risk at a colonized surface; N, not transferable in current form. Apoptotic cell clearance *programs* that require IL-4 or IL-13 for repair (52) are marked , sbecause those cytokines are already elevated in polyp tissue and are themselves the target of approved biologics (53, 54).

Bold values highlight key biological molecules, signaling pathways, and functional targets that form the core research elements of the proposed efferocytic failure cascade in chronic rhinosinusitis with nasal polyps, and also mark annotated feasibility letters D, T, and R within square brackets.

## 2 Efferocytosis is not general phagocytosis, and why that matters here

The two processes converge on Rac1-dependent cytoskeletal work and on phagosome maturation, yet they diverge at three levels that are decisive for this argument.

Recognition differs. Efferocytosis begins with phosphatidylserine (PS) displayed on the outer leaflet of a dying cell, read either directly, by receptors such as TIM-4 and CD36, or indirectly through soluble bridges, notably Gas6 and PROS1 for MerTK and AXL. Microbial uptake instead depends on opsonins and on pattern recognition (6, 8).

Output differs. Ingesting a microbe licenses an oxidative, cytokine-producing response, whereas ingesting a corpse elicits transforming growth factor β, interleukin (IL)-10 and prostaglandin E2, and TAM receptor *signaling* induces SOCS1 and SOCS3, which dampen Toll-like receptor and cytokine cascades (9). Efferocytosis is therefore immunologically silencing by design.

Consequence differs. Material derived from the ingested cell re*programs* the phagocyte through liver X receptor (LXR) and peroxisome proliferator-activated receptor (PPAR) *signaling* and licenses further rounds of uptake (10, 11). Clearance is thus an input that builds resolving capacity, not merely an act of disposal.

Two consequences follow for how evidence should be handled. First, impaired bacterial phagocytosis is permissive but not diagnostic. It is compatible with a lesion in shared machinery, yet it does not *localize* a defect to PS recognition, and reduced PS receptor function can coexist with preserved microbial uptake. The frequently quoted finding that sinonasal macrophages in chronic rhinosinusitis are M2-skewed and take up *Staphylococcus aureus* poorly (12) is therefore treated here as circumstantial rather than as demonstration of eosinophil clearance failure. Second, the discriminating experiment is a paired assay within one macrophage preparation, autologous apoptotic eosinophils against *labeled* opsonized bacteria, read alongside surface receptor levels. On the same reasoning we exclude myeloid autophagy deficiency, which aggravates eosinophilic chronic rhinosinusitis (13), from the evidence base for efferocytic failure, because autophagy acts principally on cargo degradation and constitutes a separate pathway. We cite it only as an indication that macrophage-intrinsic *programs* shape eosinophilic disease.

## 3 Graded evidence in type 2 airway disease and in CRSwNP

Tier i, causal evidence *in vivo*. Mice lacking Mer resolve ovalbumin-induced airway inflammation slowly, remain hyperresponsive, and accumulate uncleared dying eosinophils; alveolar macrophage uptake of apoptotic cells depends on Mer, and its absence amplifies the inflammatory response to apoptotic cells (14). Clearance is therefore a determinant of resolution rather than a bystander (15).

Tier ii, functional evidence in human airway disease. Airway macrophages in severe asthma express less AXL, *recognize* apoptotic cells less efficiently, and permit accumulated apoptotic cells to suppress IL-10 release (16). In obese asthma, sputum macrophage efferocytosis is about 40 per cent lower than in non-obese asthma, and the efferocytic index correlates with baseline glucocorticoid receptor α expression and with dexamethasone-induced MKP-1 (17). Human data therefore already couple clearance capacity to steroid responsiveness. Free granules released from poorly cleared eosinophils illustrate the tissue consequence (18).

Tier iii, receptor-level evidence in CRSwNP. AXL is overexpressed on sinonasal macrophages in proportion to severity and to postoperative recurrence (19), and among 150 patients followed after endoscopic sinus surgery, aberrant expression of TYRO3, AXL and MER tracked recurrence (20). These studies *localize* receptor dysregulation to the target tissue but do not measure clearance.

Tier iv, circumstantial evidence (12).

Downstream consequences are consistent across tiers. Eosinophils that are not removed in time lyse rather than resolve, releasing granule proteins, extracellular traps and Charcot-Leyden crystals (21), a sequence accentuated where *S. aureus* is present at epithelial barrier defects (22), and the crystals then activate the NLRP3 inflammasome and drive IL-1β (23). Failed clearance therefore does more than prolong inflammation, since it creates a fresh, damage-associated input that feeds back into the type 2 circuit (**Figure 1**). The absent tier, a direct clearance assay in primary polyp macrophages, is the decisive test.

## 4 A Cascade-anchored, receptor-resolved model of the polyp macrophage

The polyp macrophage compartment is M2-expanded, which is usually read as pro-resolving because alternatively activated cells support repair (24, 25). We propose replacing that label with position along the efferocytic cascade. This single move resolves both the *polarization* paradox and the apparent fragmentation of reported subsets (**Table 1**).

### 4.1 Lesion 1, loss of the clearance-competent subset

Human macrophages clear early apoptotic cells efficiently only after M2c *polarization* with MERTK induction, and IL-10 is required for that induction (26). In eosinophilic CRSwNP, IL-10-producing M2 cells are reduced even though M2 markers are raised overall, and their frequency varies inversely with symptom scores, polyp size and total inflammatory cell counts (27); IL-10 dysregulation recurs across cohorts as a property of this compartment (28). Because IL-10 both induces and is produced by MerTK-competent cells, its deficiency predicts selective depletion of an M2c-like, MerTK-high population while IL-4-driven M2a cells expand. The defect is compositional rather than numerical, which explains why M2 marker positivity and clearance competence come apart.

### 4.2 Lesion 2, repertoire skewing and uncoupling of expression from ingestion

MerTK engages PS through Gas6 or PROS1, dimerizes, autophosphorylates and drives PI3K, AKT and Rac1-dependent cup formation (29). A second arm matters equally: MerTK *signaling* raises SERCA2 through ERK, lowers cytosolic calcium, suppresses CaMKII and thereby preserves the cytosolic, non-phosphorylated pool of 5-lipoxygenase from which lipoxins and resolvins are generated (30). One receptor therefore drives ingestion and also selects the mediator class that follows, while TAM-induced SOCS proteins restrain concurrent innate *signaling* (9). AXL is regulated differently and is *favor*ed under inflammatory and IL-4-conditioned states (19, 31); in CRSwNP it is abundant and promotes M2 *polarization*, severity and recurrence (19), behaving more as a *polarization* and survival receptor than as a clearance receptor. An AXL-high, MerTK-low repertoire is therefore predicted, and the ratio rather than either receptor alone is the informative index.

TIM-4 makes the uncoupling explicit, since it tethers PS but has no catalytic domain and requires a partner kinase to complete ingestion. In CRSwNP, TIM-4 is increased on sinonasal macrophages, raises TGF-β1 through reactive oxygen species, p38 and Egr-1, drives epithelial to mesenchymal transition, and its neutralization reduces polypoid lesions (32). Prolonged tethering without ingestion converts a clearance receptor into a profibrotic sensor, which is what limiting MerTK predicts. The same logic applies to mediator output. ALOX15-positive M2 cells expand in eosinophilic CRSwNP and remodel epithelium through CCL13 (33), yet ALOX15 is normally induced by efferocytosis itself through LXR activation (11, 34). Abundant ALOX15 without efferocytic input therefore marks a lipoxygenase uncoupled from clearance and biased toward remodeling.

### 4.3 Lesion 3, an execution and cargo-handling block

Cup closure requires timed phosphoinositide turnover and Rac1 cycling. TIPE2, a phosphoinositide transfer protein that binds and inhibits Rac (35), rises in alternatively activated macrophages of CRSwNP in proportion to eosinophilia and severity (36), while INPP4A, which hydrolyses PI(3,4)P_2_, is deficient and *favor*s pathological M2 *polarization* (37). Both converge on one module and predict failure downstream of intact binding, a state in which receptor staining appears normal while uptake does not occur. HMOX1-high M2 cells that track eosinophil chemokines (38) fit a cargo-stress signature rather than a resolving one.

CD36 links this lesion to the type 2 network. It reads both PS and oxidized phospholipids and cooperates with integrins through Src family kinases. Its loss delays clearance of apoptotic alveolar cells and aggravates inflammation while lowering IL-4, IL-9, IL-13 and arginase 1 (39), coupling an efferocytic receptor to the very cytokines that define eosinophilic CRSwNP. In an oxidant-rich polyp, occupancy of CD36 by oxidized phospholipids offers a plausible route to engagement without productive clearance.

## 5 Reconciling the sheddase discrepancy: from shedding to surface availability

Section 4 requires a mechanism that lowers available surface MerTK. Cleavage by ADAM17, downstream of reactive oxygen species, protein kinase Cδ and p38, is the canonical route (40). The single CRSwNP tissue dataset, however, reports ADAM17 messenger RNA and protein to be higher in inferior turbinate than in polyp (41), the opposite of a naive prediction. We regard this as informative rather than fatal, for four reasons.

First, bulk tissue mixes cellular sources. Turbinate mucosa is dense in epithelium, glands and vessels, whereas polyp tissue is edematous and comparatively poor in cells, so whole-tissue normalization can invert a signal restricted to macrophages. Second, and more fundamentally, the amount of ADAM17 protein is a weak surrogate for sheddase activity, which is set after translation by trafficking through iRhom2, by TIMP3, by phosphorylation of the cytoplasmic tail, by substrate presentation and by local PS exposure. Activity can rise where protein does not. Third, shedding is not exclusive to ADAM17, since ADAM10, measured in the same study, matrix metalloproteinases and granulocyte-derived proteases abundant in polyps can also cleave TAM ectodomains. Fourth, surface availability can fall without any cleavage, through the transcriptional routes described in Sections 4.1 and 4.2 and through altered receptor trafficking.

We therefore restate the model as reduced surface receptor availability, of which shedding is one route, and we identify this as the least supported element of the model. The decisive measurements are soluble MER and AXL in nasal lavage normalized to total protein, paired with surface MerTK on CD68-positive macrophages to yield a surface to soluble ratio, *in situ* sheddase activity, and macrophage-restricted transcriptomics. Soluble MER already behaves as a measurable index of MerTK-dependent resolution after lung transplantation (42), so the readout transfers directly.

## 6 Why raised pro-resolving mediators cannot rescue defective clearance

Endogenous pro-resolving mediators normally terminate inflammation, and these pathways appear disturbed in chronic rhinosinusitis (43). Tissue lipidomics show resolvin D2 raised in CRSwNP and in chronic rhinosinusitis without polyps relative to controls, with lipoxin A4 highest in CRSwNP (44). Since specialized pro-resolving mediators (SPMs) are known to enhance clearance, an obvious objection arises: raised SPMs should already have corrected any clearance defect. Four explanations follow from receptor biology, and they are not mutually exclusive.

First, MerTK is the effector node through which much SPM action passes. In aged animals, resolvin D1 restored clearance largely by preventing the fall in MerTK, and mice bearing a cleavage-resistant receptor were protected (45). A mediator cannot act through a receptor that has been shed or never induced, so where the MerTK-competent subset is depleted the effector arm is structurally missing.

Second, the circuit runs in both directions, which makes the lesion self-reinforcing. MerTK *signaling* sustains the cytosolic 5-lipoxygenase pool required for SPM synthesis (30), and MerTK cleavage limits pro-resolving mediator biosynthesis while aggravating inflammation (46). Receptor loss therefore degrades the ratio of pro-resolving to pro-inflammatory products even when absolute SPM pools appear normal or high.

Third, bulk lipidomics quantify pools, not *signaling*. A tissue concentration says nothing about cellular source, spatial proximity to macrophages, receptor occupancy, or balance against leukotrienes. A high absolute value is fully compatible with an inadequate ratio and with compensatory overproduction upstream of a blocked executor, a state of resolution resistance.

Fourth, enzyme and product may be uncoupled. ALOX15 is plentiful in eosinophilic CRSwNP but is carried by a remodeling-biased subset (33) and, deprived of efferocytosis-driven LXR input (11, 34), substrate flux may *favor* remodeling products over lipoxins.

This yields a stratified and falsifiable prediction: SPM treatment of polyp explants will restore clearance only in specimens retaining sufficient surface MerTK. A first pilot of pro-resolving treatment in human polyp tissue provides the platform for that test (47).

## 7 Pro-efferocytic therapy filtered through the sinonasal barrier

### 7.1 Existing therapy may already depend on this pathway

Glucocorticoids induce eosinophil apoptosis, but they also raise macrophage clearance of apoptotic cells in a Mer-dependent manner (48), an effect linked to LXR and PPAR-δ *signaling* (49). Steroids therefore require a functioning executor downstream, which predicts hyporesponsiveness where macrophage MerTK is intrinsically low. The human coupling of efferocytic index to glucocorticoid responsiveness in asthma (17) makes this biomarker hypothesis directly testable in CRSwNP.

### 7.2 Which cross-disease strategies can transfer to a colonized barrier

Efferocytosis is now druggable in principle, with CD47 and SIRPα blockade and TAM-directed agents in early clinical development (50), and enhancing clearance is proposed for chronic inflammatory disease on the understanding that the process is dual-natured and that interventions must be disease-specific (51). Three constraints specific to the sinonasal mucosa should be applied explicitly rather than assumed away.

The first is antimicrobial risk. Because TAM *signaling* induces SOCS1 and SOCS3 and broadly restrains innate responses (9), supraphysiological agonism at a surface colonized by *S. aureus* with documented barrier defects (22) could impair bacterial defense. Approaches that prevent receptor loss, such as sheddase inhibition or cleavage-resistant receptors, are conceptually safer than global agonism, although ADAM17 inhibition must be balanced against loss of ADAM17-dependent ligands required for epithelial repair.

The second is delivery. Systemic CD47 blockade is limited by hematological toxicity, whereas the sinus cavity permits topical sprays, exudative transport and drug-eluting implants, with postoperative access for repeat sampling. Mucus, biofilm and polyp oedema are the corresponding penetration problems and constitute the principal formulation question.

The third is compatibility with the local cytokine milieu. Repair *programs* that require IL-4 or IL-13 alongside apoptotic cells (52) cannot be imported into tissue in which those cytokines are already high and are themselves the target of approved biologics (53, 54). Conversely, IL-4 receptor α blockade may shift the repertoire away from an AXL-high M2a state toward MerTK-competent cells, a prediction testable in treated patients. Within these limits, hydroxychloroquine, which restores MerTK and Gas6-dependent clearance in a lupus model (55), IL-10-directed metabolic reprogramming (56), CD47-blocking antibodies that restore phagocytosis in atherosclerosis (57), and apoptotic cell-based tolerization, which reduced eosinophil recruitment and type 2 cytokine production in murine allergic airway disease (58), remain proofs of principle whose sinonasal feasibility is unestablished.

### 7.3 The opposite therapeutic direction

Balance requires recording that these same receptors are targeted for inhibition elsewhere. Because MerTK-dependent clearance restrains innate activation and supports tolerance, an antibody that selectively blocks it causes apoptotic cells to accumulate within *tumor*s, triggers a type I interferon response and synergizes with checkpoint blockade (59), and loss of MerTK in *tumor* leukocytes likewise limits *tumor* growth (60). The direction of benefit is therefore set by tissue context rather than by the receptor, which is exactly why efficacy in artery or transplanted lung cannot be assumed at a colonized mucosa. The oncology *program* nonetheless supplies selective antibodies and inhibitors with which the direction of effect in polyp tissue can be tested. We therefore present pro-efferocytic therapy in CRSwNP as a hypothesis for explant testing rather than as a treatment proposal.

## 8 Limitations and falsifiable predictions

This is a hypothesis-generating synthesis. No functional efferocytosis assay in primary polyp macrophages with autologous apoptotic eosinophils has been published; the CRSwNP receptor data are associative (19, 20); the tissue sheddase dataset remains discordant (41); and the M2a and M2c nomenclature is an imperfect proxy for states *in situ*. Two caveats deserve emphasis. Causal evidence remains confined to murine lower airway models (14), whereas the sinonasal mucosa differs in microbial exposure, mucus and epithelial architecture. Moreover, almost all polyp tissue is obtained from corticosteroid-exposed patients, and since glucocorticoids augment Mer-dependent clearance (48), existing receptor datasets may understate the defect rather than exaggerate it. Efferocytic failure is also not the only candidate explanation, since fibrin deposition driven by innate type 2 inflammation contributes to polyp formation independently of clearance (61). Our claim is additive rather than exclusive: failure to induce eosinophil death and failure to remove dead eosinophils are separable, and can be distinguished by measuring apoptotic index and macrophage uptake in the same specimen.

Three findings would refute the model: polyp macrophages that ingest autologous apoptotic eosinophils normally; surface MerTK that is preserved and unrelated to eosinophilia and recurrence; or restoration of clearance by pro-resolving mediators irrespective of baseline surface MerTK, which would place mediator action outside the hierarchy proposed in Section 6. Four experiments follow: paired apoptotic cell and bacterial uptake assays, reporting binding and internalization separately; single-cell and spatial profiling (62) to quantify macrophage-restricted receptors and the AXL to MerTK ratio against eosinophil cytolysis; soluble receptor measurement with surface to soluble ratios (42); and explant intervention stratified by baseline surface MerTK (47).

## 9 Conclusion

Persistent eosinophilia in CRSwNP has been attributed to recruitment and to delayed death. We propose a third mechanism lying upstream of lipid mediator biology: failure of macrophage efferocytosis, arising from loss of an IL-10-dependent, MerTK-competent subset, from a receptor repertoire that tethers without ingesting, and from an execution block at the phosphoinositide and Rac1 module. The proposal is deliberately narrower than a general claim of macrophage dysfunction, is separated from general phagocytic defects and from mediator-centered accounts, and specifies the measurements that would establish or refute it.
